# Anxiety, depression, and quality of life in mothers of children with congenital Zika syndrome: Results of a 5-year follow-up study

**DOI:** 10.1590/0037-8682-0627-2021

**Published:** 2022-04-08

**Authors:** Sheila Jaqueline Gomes de Oliveira, Carolina Santos Souza Tavares, Victor Santana Santos, Hudson P. Santos, Paulo Ricardo Martins-Filho

**Affiliations:** 1 Universidade Federal de Sergipe, Programa de Pós-Graduação em Ciências da Saúde, Aracaju, SE, Brasil.; 2 Universidade Federal de Sergipe, Laboratório de Patologia Investigativa, Aracaju, SE, Brasil.; 3 Universidade Federal de Alagoas, Núcleo de Epidemiologia e Saúde Pública, Arapiraca, AL, Brasil.; 4 University of North Carolina at Chapel Hill, Biobehavioral Laboratory, School of Nursing, Chapel Hill, NC, USA.

Dear Editor

From 2014 to 2017, an outbreak of Zika virus (ZIKV) infection spread in the Americas and Pacific region, and was later linked to congenital microcephaly and neurological disorders in children. Children with congenital microcephaly have severe abnormalities in the central nervous system, including brain dysgenesis and intracranial calcifications consistent with intrauterine infection. The spectrum of anomalies found in children with presumed or laboratory confirmed ZIKV infection is known as congenital Zika syndrome (CZS)^1^.

In 2016, we published the first report evaluating nine mothers of infants with CZS born in a public maternity in Sergipe state, Brazil, which had one of the highest rates of ZIKV infection in the country. This study showed high levels of anxiety and low scores of psychological well-being among mothers during the first 24 h after birth^2^. In 2017, a second study showed that these mothers continued to have high levels of anxiety and low quality of life during the first year of their children’s lives^3^. As functional limitations, co-occurring clinical conditions, and long-term dependence of children with CZS can lead to a long-term negative impact on maternal quality of life and increased risk of adverse mental health outcomes; we evaluated anxiety levels, depressive symptoms, and quality of life of these mothers in a 5-year follow-up study.

To complement the data available from the 24 h and 1-year post-birth previously reported, we assessed anxiety and depression symptoms at 5-years post-birth using the State Anxiety Inventory (SAI) and the Beck Depression Inventory (BDI), respectively. The interviews were conducted in August 2021. A cut-off point of 39 was used to detect clinically significant symptoms of anxiety. BDI scores of 10 - 18, 19-29, and 30-63 were used to distinguish mild to moderate depression, moderate to severe depression, and severe depression, respectively. Quality of life was assessed using the short version of the World Health Organization Questionnaire (WHOQoL-BREF).

The results are presented as median (25th [Q1]-75th [Q3] percentiles). We used the Friedman test with post-hoc Conover to compare scores 24 h after birth, during the first year, and at 5-year follow-up. The level of statistical significance was set at α = 0.05, and all statistical tests were two-tailed. The analyses were performed using the statistical software JASP (Version 9·1·0; Amsterdam, The Netherlands; http://jasp-stats.org/).

During the 5 years of follow-up, one child with CZS died and eight mothers who had been evaluated in 2016 and 2017 were analyzed in this study. The mothers’ ages ranged from 22 to 45 years. Five years after the children were born, all mothers had clinically significant symptoms of anxiety. The median SAI scores in the in-hospital evaluation, during the first year after birth, and at 5-year follow-up were 38.5 (34.0-44.8), 47.5 (41.3-49.0), and 47.0 (43.8-48.5), respectively. We found higher levels of anxiety at 5-year after birth compared to the in-hospital evaluation (p = 0.030). Three (37.5%) mothers had mild to moderate depression at 5-year follow-up, and the median BDI score of the entire sample was 8.0 (7.0-10.0). The median global quality of life score was 59.7 (55.9-63.8). However, no differences in quality of life or depressive symptoms were observed over time (Table 1).

**TABLE 1:** Changes in quality of life, depressive symptoms, and anxiety levels among mothers of children with congenital zika syndrome.

Outcomes	In-hospital	1-year	5-year	p-value			
				Friedman	Conover’s post-hoc test		
					In-hospital vs. 1-year	In-hospital vs. 5-year	1-year vs. 5-year
**Quality of life**							
Physical	64.3 (56.2-73.2)	64.3 (46.4-70.6)	64.3 (59.8-67.9)	0.733	0.697	0.697	0.441
Psychological	60.4 (57.3-66.7)	58.4 (45.8-66.7)	58.3 (54.2-62.5)	0.381	0.308	0.207	0.795
Social	66.7 (58.3-79.2)	58.4 (47.9-77.1)	66.7 (56.2-75.0)	0.485	0.254	0.441	0.697
Environmental	56.3 (49.2-67.2)	51.6 (45.2-63.3)	50.0 (46.1-57.1)	0.250	0.326	0.120	0.535
Global	61.6 (56.4-67.0)	56.6 (49.8-63.7)	59.7 (55.9-63.8)	0.417	0.334	0.232	0.806
**Depressive symptoms**	5.0 (0.0-9.0)	11.0 (5.8-16.5)	8.0 (7.0-10.0)	0.159	0.077	0.458	0.271
**State anxiety**	38.5 (34.0-44.8)	47.5 (41.3-49.0)	47.0 (43.8-48.5)	0.044*	0.096	0.030*	0.535

Statistical significance was set at p < 0.05. Data are reported as median and interquartile range (Q1-Q3).

This study showed that mothers of children with CZS had a long-term course of clinically significant levels of anxiety symptoms. In addition, quality of life scores remained low over time. Previously, we reported that these mothers had significantly lower scores in the psychological domain of WHOQoL-BREF than mothers of healthy neonates and the lack of maternal psychosocial support and counseling during the first year of follow-up^2^ ^,^ ^3^. Poor mental health in mothers of children with CZS may also be associated with the health demands and care of the child^4^, lack of social support^5^ ^,^ ^6^, changes in occupational roles^7^, and stigmatization of the disease^4^. A previous study found a relationship between increased levels of anxiety and lower levels of acceptance of self and life among these mothers^8^. In addition, the lack of health rehabilitation services, especially in the poorest regions, Constitutional Amendment 95 that reduces public investment in health, reformulations in social cash transfer programs, general impoverishment of the Brazilian population, increase in unemployment and inflation rates, and the emergence of the coronavirus disease-2019 pandemic are critical macrostructural factors that affect the living conditions of families and the care of children with disabilities.

The mental health status of mothers of children with CZS has been neglected. We call for attention and action to provide these mothers with psychological support, social assistance, and counseling, as well as an employment and income generation program, especially for mothers in situations of higher socioeconomic vulnerability. Furthermore, it is necessary to guarantee continuous and specialized care for children with CZS, which must be articulated within the national health system.
